# Aptamers for DNA Damage and Repair

**DOI:** 10.3390/ijms18102212

**Published:** 2017-10-22

**Authors:** Maureen McKeague

**Affiliations:** Department of Health Sciences and Technology, ETH Zürich, Schmelzbergstrasse 9, 8092 Zurich, Switzerland; maureen.mckeague@hest.ethz.ch; Tel.: +41-44-632-3282

**Keywords:** aptamer, DNA damage, DNA repair, in vitro selection, SELEX, mutation, therapeutics

## Abstract

DNA is damaged on a daily basis, which can lead to heritable mutations and the activation of proto-oncogenes. Therefore, DNA damage and repair are critical risk factors in cancer, aging and disease, and are the underlying bases of most frontline cancer therapies. Much of our current understanding of the mechanisms that maintain DNA integrity has been obtained using antibody-based assays. The oligonucleotide equivalents of antibodies, known as aptamers, have emerged as potential molecular recognition rivals. Aptamers possess several ideal properties including chemical stability, in vitro selection and lack of batch-to-batch variability. These properties have motivated the incorporation of aptamers into a wide variety of analytical, diagnostic, research and therapeutic applications. However, their use in DNA repair studies and DNA damage therapies is surprisingly un-tapped. This review presents an overview of the progress in selecting and applying aptamers for DNA damage and repair research.

## 1. Introduction

**Figure 1:**
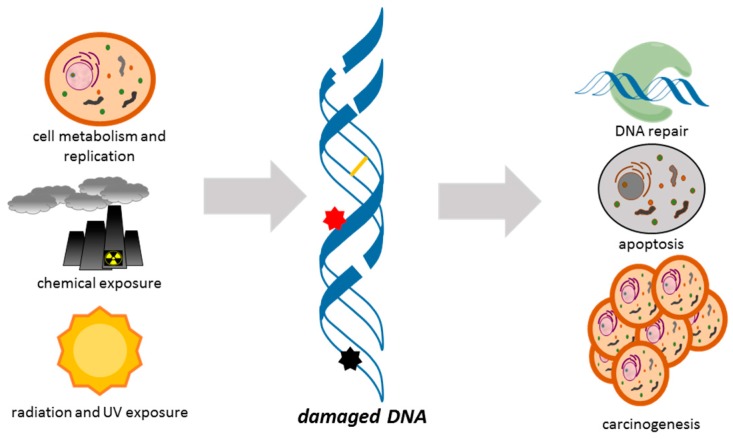
Our DNA is damaged by normal cell processes, contaminants in our food and environment, radiation and ultraviolet light. Damage may include strand-breaks, crosslinks (yellow line in DNA), or adducts (black and red stars). If not repaired, DNA damage can lead to cell death or heritable mutations and cancer.

While DNA was originally considered an extremely stable molecule, Tomas Lindahl determined that the nucleobases of DNA react slowly with water. This insight led him to discover the molecular machinery by which cells repair damaged DNA and won him, along with Aziz Sancar and Paul Modrich, the Nobel Prize in Chemistry in 2015 [1,2,3]. Indeed, our DNA is damaged on a daily basis by radiation, ultraviolet light and contaminants in our food and in our environment. DNA damage can lead to heritable mutations and the activation of proto-oncogenes. DNA damage is therefore a critical risk factor in cancer, aging and heritable diseases [4], and is the underlying basis for most frontline cancer therapies [5,6] (Figure 1).

Cells have a number of strategies to detect (e.g., damage checkpoints) and deal with (i.e., repair pathways) damage to DNA. If such detection and repair mechanisms are impaired, cells may experience genomic instability, apoptosis or senescence, and furthermore, predispose organisms to immunodeficiency, neurological disorders and cancer. Antibodies have been widely used as the molecular recognition platform of choice for the detection of DNA adducts and repair signaling and activation [7,8]. Over the past several decades, their use in DNA damage and repair research has facilitated the discovery of and insight into the mechanisms by which cells respond to DNA damage and initiate repair.

More recently, the nucleic acid analogues to antibodies, known as aptamers, have emerged. Since their discovery, aptamers have been compared to antibodies due to their similar ability to bind to specific targets. However, aptamers offer several broad advantages over antibodies as molecular recognition molecules (reviewed elsewhere [9,10]). Antibodies must be developed in vivo, whereas aptamers are composed of oligonucleotides which can be selected in vitro (using a process termed the systematic evolution of ligands by exponential enrichment, SELEX). As a result, aptamers can be selected against DNA-damaging toxins or the resulting lethal DNA adducts that are challenging for antibody generation. Furthermore, the nucleic acid composition affords aptamers with the ability to reversibly change conformation, making it possible to develop aptamers in conditions with varying pH, temperatures and ionic strengths that would cause antibodies to be irreversibly denatured. Finally, aptamers can be chemically modified during their synthesis to increase shelf life and nuclease resistance, as well as impart different chemical functionality (e.g., fluorescence and electrochemical properties) [11].

The unique combination of aptamer qualities listed above has led to a surge in the application of aptamers for analytical and diagnostic detection, therapeutics and drug delivery, intracellular imaging, and for gene regulation and control. With this increased interest in aptamers, coupled to a growing concern of the quality of commercial research antibodies [12], it is surprising that the application of aptamers to the study and detection of DNA damage and repair processes is limited. This review covers the aptamers that have been selected for DNA damage and repair proteins to-date and discusses the challenges in their selection and potential applications.

## 2. Aptamers for Damaged DNA

Damage to DNA includes oxidation, depurination or depyrimidation, single-strand or double-strand DNA breaks, deamination or alkylation. In this section, aptamers that can be potentially used to detect these types of DNA damage are highlighted.

### 2.1. Guanine Oxidation

**Figure 2:**
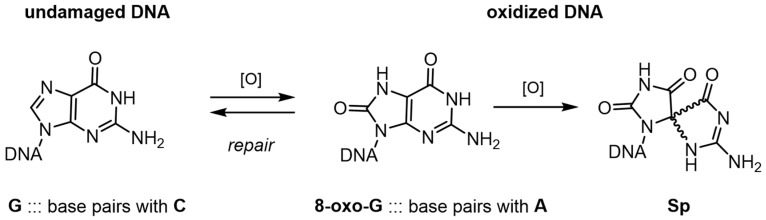
When guanine is oxidized, forming 8-oxoguanine (8-oxo-G), the resulting preferential basepairing to A ultimately leads to a G to T tranversion mutation. Further oxidation results in the spiroiminodihydantoin (Sp) adduct diastereomers, for example, which are highly destabilizing to the DNA duplex.

The most abundant oxidatively-damaged base is 8-oxoguanine (8-oxoG.) When occurring in the genome, mismatched pairing of 8-oxoG with adenine results in G to T transversion mutations [13]. G to T mutations are often observed in oxidative stress-associated diseases, such as cancer, atherosclerosis, diabetes and pathologies of the central nervous system, as well as during aging. Therefore, it is not surprising that this damaged-nucleobase has been the focus of recent detection strategies [14], and the target of many aptamer-related selections. 8-oxoG can be further oxidized, leading to the formation of a variety of products, including the spiroiminodihydantoin (Sp) lesion. Unlike the 8-oxoG lesion, the Sp lesion is not planar. Instead, it is shaped like a propeller. Given this unusual shape, both Sp lesions strongly destabilize the DNA duplex [15] (Figure 2).

The first example of an aptamer that recognized DNA damage was reported in 1998 by Rink et al. Following ten rounds of conventional SELEX using an 8-oxodG affinity matrix, a specific RNA aptamer (Clone R10-B35) that exhibited highly specific binding to 8-oxodG compared to dG and other nucleosides was selected [16]. Using the electrophoretic mobility-shift assay (EMSA), the authors demonstrated that the aptamer bound to a single-strand DNA sequence containing a 3′ terminal 8-oxodG with an apparent dissociation constant (*K*_d_) of 270 nM. Surprisingly, the RNA aptamer also recognized 8-oxodG present in the center of a 19 nt ssDNA with an apparent *K*_d_ of 2.8 µM.

Almost ten years later, in 2009, the first DNA aptamer that recognized the oxidative lesion 8-oxodG was reported. To select these aptamers, Miyachi and co-workers used guanosine-monophosphate as an analog of 8-oxodG (due to the difficulty in immobilizing sufficiently high concentrations of 8-oxodG). The highest affinity aptamer that emerged from their selection was capable of binding to the free nucleoside with a *K*_d_ of 0.1 µM [17].

In 2012, an aptamer for 8-oxoG (the free base) was rationally designed. To achieve this, the thermal stability of nine 8-oxodG-containing hairpin DNA triplexes were compared. Next, the 8-oxoG moiety was removed from the two most stable triplexes. As a result, the abasic site allowed for the free oxidized base to bind in a specific manner [18]; thus creating a “rationally designed aptamer”. The same group also tested several aliphatic side chain modifications at the abasic site to improve binding specificity. Introduction of a β-alanine side chain allowed selective binding of the 8-oxoG nucleobase with a dissociation constant of 5.5 µM [19].

Finally, in 2015, the Burrows group employed the recently described “structure-switching” SELEX [20] to isolate DNA aptamers for several products of guanine oxidation, including 8-oxodG and its nucleobase (8-oxo-G), the dSp nucleoside diastereomers: (−),-(*R*)-dSp and (+),-(*S*)-dSp and one of the Sp nucleobase enantiomers: (−),-(*R*)-Sp. The DNA aptamers resulting from this work bound to their respective targets with *K*_d_ values in the low nanomolar range, with the exception of the aptamer for 8-oxo-dG, which bound in the micromolar range (Table 1) [21].

### 2.2. Guanine Alkylation

Adduct formation is the result of covalent binding between reactive electrophilic substances and nucleophilic sites (ring nitrogens and exocyclic oxygen atoms) of DNA bases. The N7 atom of guanine is the most vulnerable site for attack by alkylating agents [34]. Other common sites of DNA alkylation include the *N*^3^ and *N*^1^ positions of adenine, as well as the *N*^3^ position of cytosine. Furthermore, even though *O*-alkyl lesions are generated to a much lesser extent than *N*-alkyl adducts, the induction of *O*^6^-alkyl-G lesions is of significant interest because *O*^6^-alkyl-G can readily mispair with thymine during DNA replication to cause mutagenic and cytotoxic biological effects.

While there are no reports of aptamers that directly bind to the adduct *N*^7^-methylguanine (m^7^dG), there are a few potential examples that might be explored in the future. As one example, Haller et al. reported the selection of RNA aptamers that bind to the methylated ribose, 7-methyl-guanosine (m^7^G) which is typically part of the 5′ cap at the ends of mRNA transcripts in eukaryotes. The authors performed conventional SELEX with the goal of isolating aptamers capable of inhibiting translation of capped mRNA transcripts. The resulting aptamers bound to m^7^G with modest affinity (0.5 µM) and high specificity, discriminating between non-methylated nucleotides by over 2000 fold. Interestingly, the presence of phosphate groups, or the identity of the purine group (e.g., adenine vs. guanine) had little effect on binding [22]. Therefore, it is possible that these aptamers may recognize the m^7^dG adduct.

As another example, Larguinho and co-workers used an RNA aptamer that binds to xanthine [35] to develop nanoprobes that preferentially detected glycidamide (GA) adducts. GA is an epoxide metabolite of the genotoxic carcinogen acrylamide, and alkylates the *N*^7^ position of guanine. Surprisingly, the authors were able to preferentially detect GA adducts compared to similar compounds (e.g., dGTP and glycidamide metabolites). While the sensor was capable of detecting the GA adducts, it was unfortunately ineffective in the presence of high concentrations of nucleotides [36].

Finally, Xu et al. developed an RNA aptamer that binds benzylguanine [23]. While benzylguanine is not a biologically-relevant DNA adduct, it is frequently used in place of *O*^6^-methylguanine for studies [37]. The best selected aptamer displayed high affinity for the target (~200 nM), but most importantly, it exhibited a 20,000 fold selectivity compared to other guanine metabolites.

Together, these examples suggest that aptamers could be selected to recognize and bind to biologically-relevant alkylated guanine adducts in the future.

### 2.3. Double-Strand Breaks

The most dangerous forms of DNA damage are double-strand breaks (DSB). DNA DSBs occur when the two complementary stands of the double helix are simultaneously broken at locations that are close enough to one another, so that base pairing and chromatin structure cannot keep the two DNA ends together. As a consequence, repair is difficult and detrimental recombination with other sites in the genome may occur [38].

Despite their importance, there have been no reports of selecting aptamers to specifically recognize the termini of DSBs. This is expected to be difficult due to the electrostatic repulsion of the sugar–phosphate backbone of DNA that would prevent single strand aptamer binding to the DNA duplex. However, there have been several efforts in the past two decades to overcome the challenges presented by binding oligonucleotides to duplex DNA.

The Maher group reported the first example in 1996. The authors applied conventional SELEX against a 21 nt homopurine/homopyrimidine duplex DNA target. Following 26 rounds of selection under conditions that increased from pH 5.0–7.4, several aptamers emerged that bound to the duplex target with modest affinity (less than 1 µM) at pH 6, and approximately 10 µM at physiological pH [24]. Importantly, these selected aptamers approach the binding affinity of a 21 nt RNA oligonucleotide that forms a canonical triple helix with the duplex. Building on the success of this SELEX approach, selection of oligonucleotides to a duplex was performed again 13 years later, this time at neutral pH and in the presence of the triplex stabilizing agent, benzoindoloquinoline (BIQ). Following only seven rounds of selection, aptamers were capable of binding a 20 bp duplex with a *K*_d_ of 43.9 nM. However, in the absence of BIQ, no binding was observed [25]. Together, these examples indicate that there are strategies to reduce the electrostatic repulsion between DNA and potential aptamers; thus, potentially enabling future approaches for aptamer-based detection of DSBs.

As a final exciting example, Srisawat et al. performed SELEX using the 3′ long terminal repeat (LTR) of human immunodeficiency virus type 1. This 325 bp DNA duplex lacks a long polypurine/polypyrimiding tract and is therefore unlikely to favor triplex formation. The goal of this work was to identify aptamers that bind to the internal region of the LTR and thereby regulate its transcription. As such, several efforts were made to promote binding of the aptamer library to the internal region. The authors found that the selected RNA aptamers had a tendency to bind to the “ends” of the dsDNA; some aptamers were specific for the 3′ end while others recognized the 5′ end of the LTR [26]. This result suggests that aptamers might be selected to recognize the damaged ends of double-strand breaks.

## 3. Aptamers for Repair Proteins

Cells have unique molecular pathways to correct common types of DNA damage. The major pathways include non-homologous end joining, homologous recombination, mismatch repair, nucleotide excision repair, base excision repair and direct repair [39]. Below, the aptamers that bind to the repair proteins that mediate these repair pathways are described. The repair mechanisms and proteins involved in each pathway have been extensively reviewed by others (see for example [40,41]); however, a short overview is also provided. To our knowledge, there are no aptamers available for the components of homologous recombination repair pathways. Furthermore, there are no aptamers that specifically interact with proteins from transcription-coupled repair (TC-NER) involving the transcription factor TFIIH. However, a recent review describes many aptamers that bind to the transcription factor, TFIIA [42]. This suggests that the TC-NER pathway may be a suitable target for future aptamer development.

### 3.1. Non-Homologous End Joining

Double-strand breaks (DSBs) are repaired by the non-homologous end joining (NHEJ) and homologous recombination repair (HR) pathways. While the HR pathways require a homologous template, the NHEJ pathway repairs DSBs by directly ligating the ends. There are at least two genetically distinct sub-pathways of NHEJ: the classical-NHEJ (C-NHEJ) and alternative-NHEJ (A-NHEJ). The C-NHEJ pathway requires at least seven different proteins [43]. Two of these proteins, Ku70 and Ku86, form a heterodimer that functions as a molecular scaffold for the other NHEJ proteins to bind and initiate repair [44,45].

The first example of aptamer selection for a repair protein was performed in 1998 to the important scaffolding protein Ku. Here, Yoo et al. performed electrophoretic mobility shift assay (EMSA)–SELEX using an RNA library against the dimeric Ku protein purified from HeLa cell extracts. Their selection yielded 18 individual aptamers, each binding to the Ku protein with dissociation constants below 2 nM [27]. Excitingly, four of the aptamers were sufficiently selective that they were able to bind to the Ku protein in crude Hela cell extracts. Furthermore, these aptamers inhibited the binding and catalytic activity of the DNA dependent protein kinase catalytic subunit (DNA-PK), which is normally recruited to broken ends of DNA by the Ku protein [27].

### 3.2. Base Excision Repair

The base excision repair (BER) pathway is responsible for repairing small, non-helix-distorting damaged bases. BER is initiated by DNA glycosylases that recognize and remove the damaged base, creating an abasic site (AP site). Next, an AP endonuclease cleaves the AP site, and DNA polymerase β (polβ) removes the resulting 5′-deoxyribose phosphate via its 5′ to 3′ nuclease activity. Finally, the gap is filled by either short-patch (polβ replaces a single nucleotide) or long-patch BER (2–10 nucleotides are newly synthesized) [46,47].

There are currently two known examples of aptamers selected to BER-related proteins. First, as a proof-of-concept study, the Beal lab selected RNA aptamers to formamidopyrimidine DNA glycosylase (Fpg). Fpg (also known as 8-oxoguanine DNA glycosylase) is found in bacteria and repairs a wide range of oxidized purines. In this work, Vuyisich et al. used conventional SELEX with Fpg isolated from *E. coli* as the target. However, the authors were interested in isolating ligand-induced binding aptamers (i.e., those that only bind to the target in certain conditions). Therefore, the selections included a range of neomycin concentrations. As a result, the emerging aptamers could only bind to the target, Fpg, in the presence of the antibiotic, neomycin. Regardless, the best aptamer displayed high affinity to this repair protein, with a reported *K*_d_ of 7.5 nM [28]. Next, in 2006, Gening and co-workers performed seven rounds of selection to uncover RNA aptamers that bind to polβ isolated from *E. coli*. Upon further characterization, these aptamers bound to polβ with *K*_d_ values as low as 290 nM. Unfortunately, the aptamers did not display high specificity, binding also to polκ with similar affinity (*K*_d_ = 410 nM). More impressively, the aptamers were able to inhibit the activity of both polymerases in primer extension assays [29], highlighting potential applications for these aptamers.

### 3.3. Mismatch Repair

Mismatches in the genome can occur due to mis-incorporation during the replication process, or as a result of chemical damage to a complementary nucleobase. If the mismatches are not removed by the proofreading activities of the replisome, then the post-replicative “Mismatch Repair System” (MMRS) is activated. For simplicity, the process in *E. coli* is described; however, homologues of all these proteins are found in eukaryotes. This process is initiated by MutS, a protein that recognizes and binds to mispaired nucleotides. MutS then works together with MutL to direct the excision of the newly synthesized DNA strand by MutH [48]. This is followed by removal of the mismatch and subsequent re-synthesis by DNA polymerases [49].

The Krylov group has been using non-equilibrium capillary electrophoresis of equilibrium mixtures (NECEEM) SELEX [50] to identify many aptamers to various repair proteins. In 2006, NECEEM was first used to select aptamers to MutS from *Thermus aquaticus*. The isolated aptamers displayed dissociation constants as low as 3.6 nM to the isolated protein [30]. Other selections by the Krylov group have focused on DNA dealkylating proteins (see below).

### 3.4. Direct Repair

The simplest form of repairing DNA damage is direct repair, because cleavage of the phosphodiester backbone is not required. As a result, highly specialized proteins are involved for each type of damage [51]. Direct reversal is primarily used for correcting damage caused by DNA alkylating agents. For example, *O*^6^-alkylguanine DNA alkyltransferase (AGT) is known to specifically reverse *O*^6^-methylguanine back to guanine. As another example, the repair protein AlkB directly repairs *N*^1^-methyladenine and *N*^3^-methylcytosine base lesions [52,53].

In 2011, the Krylov group selected DNA aptamers using the NECEEM SELEX platform to the AlkB protein isolated from *E. coli*. The resulting aptamers displayed *K*_d_ values in the nanomolar range (as low as 20 nM) [31]. Later, these aptamers were shown to also inhibit AlkB activity by binding through an allosteric mechanism [54]. Next, the Krylov group was interested in obtaining aptamers to the human repair protein homologue, AlkB homologue 2 (ABH2). Original attempts failed due to challenges with the target (including instability and high positive charge). However, in 2014, NECEEM was coupled to emulsion PCR to efficiently amplify potential sequences. As a result, aptamers were isolated within three rounds that bound specifically to ABH2 with *K*_d_ values as low as 85 nM [32].

## 4. Aptamers That Recognize Mutated Gene Products

If DNA adducts are not repaired, mutations accumulate in the genome. When these mutations occur in oncogenes or tumor suppressor genes, it is possible that the mutations confer a growth advantage (driver mutations), thus resulting in the promotion of cancer [55]. Hot spot mutations may arise from either potentially damage-prone or repair-inaccessible locations in the genome. Ongoing research aims to determine the exact mechanism behind this selection preference [56]. Such mutations occur in tumor samples more frequently than the background [57], and have been identified in several genes. As a result, these mutated gene products provide some of the best-studied targets for chemotherapy [57], which explains why there have been some efforts towards isolating aptamers for these cancer driver gene products.

Codon 12 of the *KRAS* gene is the most frequently mutated codon in human cancers. As a result, many aptamers have been generated to mutant KRAS proteins and peptides [58,59]. In the most recent example, an RNA aptamer was generated that specifically bound to a mutant KRAS protein with a point mutation in codon 12 (KRAS^V12^). Excitingly, binding to the wild-type KRAS was more than 50 fold lower than the mutant [33]. A second example is the *p53* gene which is considered the “guardian of the genome”. *p53* is lost or mutated in about half of human cancer cases [60,61]. The single amino acid substitution p53R175H is one mutation which abolishes p53 function. In 2015, Chen et al. were able to isolate an RNA aptamer that binds to the p53 mutant p53R175H. Remarkably, this RNA aptamer (p53R175H-APT) also displayed a significantly stronger affinity to p53R175H than to the wild-type p53 in both in vitro and in vivo assays [62].

## 5. Selection Challenges and Considerations

**Figure 3:**
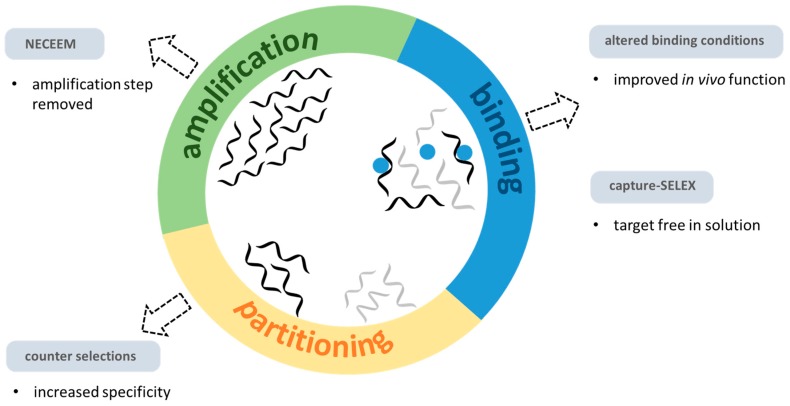
Conceptual representation of classic Systematic Evolution of Ligands by EXponential enrichment (SELEX) and important modifications. Classic SELEX consists of iterative rounds of binding, partitioning and PCR amplification. Single-stranded DNA or RNA libraries are incubated with the target-of-interest (blue circles). A partitioning step removes non-specific sequences (light grey strands). PCR amplification is then used to make multiple copies of the selected sequences (dark grey). Modifications to the classic SELEX process to isolate aptamers for DNA damage and repair targets include: the use of “capture-SELEX” for small molecules allowing them to be selected without immobilization; altered binding conditions to improve binding to strand breaks and improving activity in vivo; rigorous counter selection to ensure binding specificity; and the use of NECEEM for difficult protein targets.

The SELEX process involves iterative rounds of in vitro binding, partitioning and amplification (Figure 3) [63,64,65]. Despite the simplicity, a major advantage of the process is the flexibility in the enrichment strategy, binding conditions and nucleic acids design and type [66,67]. Due to this flexibility, aptamers have been selected to a wide range of targets, including whole cells, viruses, proteins and small molecules [68]. For reviews on the many modifications and improvements to the SELEX procedure over the past 25 years, see [69,70,71]. Here, conditions specific to DNA damage and repair targets are highlighted.

### 5.1. DNA Adducts

The nucleobases of DNA have molecular weights ranging from approximately 110–150 g/mol. Nucleosides range from 240–285 g/mol, and nucleotides are around 500 g/mol. As a result, the selection targets for DNA damage aptamer libraries are very small, and therefore pose some of the same challenges as small molecule SELEX. Several reviews and methods highlight the conceptual and technical challenges in isolating aptamers to targets of less than 1000 g/mol [11,72]. This explains, in part, the relatively small number of different DNA adduct aptamers as compared to repair proteins, and is consistent with the general trend of fewer small molecule aptamers as compared to aptamers to large targets such as proteins and even cells [68].

The biggest potential break-through in addressing the challenges associated with small molecule aptamer selection was the development of Capture-SELEX, which yields structure-switching aptamers [73]. This method circumvents the needs to immobilize small molecules on a solid-support and further introduces a selection pressure for the selected aptamers to undergo a large structural rearrangement upon binding to the target. This feature is often desired in development detection applications with aptamers [74]. As a result, future aptamer selection efforts to damaged nucleobases and nucleosides should make further use of the Capture-SELEX strategy.

### 5.2. Strand Breaks

Overcoming electrostatic repulsion of the sugar–phosphate backbone of DNA typically requires the alteration of binding conditions in the selection of aptamers. This may include high concentrations of cations (to shield the charge), lowering the selection pH or adding triplex-stabilizing agents. However, a relatively unexplored strategy is the incorporation of non-natural nucleotides. One potential option would be the use of peptide nucleic acids (PNA), where the negatively charged phosphate backbone is replaced by a neutral amide backbone [75]. As a result, PNA can selectively bind and invade the DNA duplex [76]. There are several examples of DNA aptamers being synthesized as PNA or developing aptamer-PNA conjugates [77]. Furthermore, the Liu lab has described an in vitro selection and amplification system for peptide nucleic acids [78]. Therefore, future efforts should evaluate the use of PNA to improve the binding affinity and specificity of detecting strand breaks.

### 5.3. Proteins

There are several methods available for selecting aptamers to protein targets; each possessing their own unique advantages and difficulties. Regardless of the method employed, the most important consideration for aptamers in applications involving DNA damage and repair is ensuring specificity. This is particularly critical for targeting both repair proteins and mutated gene products. In the selections described for mutated genes (Section 4), counter selection rounds were imperative to ensure that the resulting aptamers did not bind to the non-mutated, wild-type proteins. Without including these counter measures, it is possible that the aptamers would also recognize and bind to the wild-type proteins. For example, in the polβ selection (Section 3.2), counter selections were not performed. As a result, the aptamers were able to additionally bind and inhibit polκ, a polymerase from a different family. Therefore, highly stringent counter selection steps should be performed to ensure the specificity of the isolated aptamers.

## 6. Promising Applications

**Figure 4:**
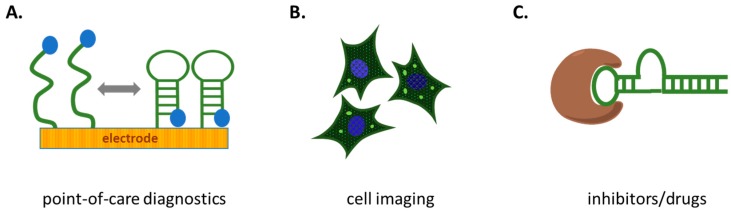
Conceptual figure highlighting potential applications of aptamers for DNA damage and repair. (**A**) Aptamers (green strands) could be incorporated into several platforms to create rapid analysis or point-of-care diagnostic kits to measured DNA lesion levels; (**B**) Aptamers combined with RNA tools such as “Spinach” could replace antibodies in cell imaging (allowing fluorescent imaging (bright dots) of damage/ repair proteins inside cells); (**C**) Highly specific aptamers (green strand) that inhibit repair proteins and polymerases (brown shape) could be used in cancer treatment and gene therapy.

There is a disproportionately large number of publications and patents describing aptamer applications compared to the number of publications describing new aptamers [79]. This trend is consistent across most areas of aptamer research including food safety [80], neuroscience [81], medicine [82] and gene control [83]. In contrast, the opposite appears to be true in the field of DNA damage and repair; there are fewer examples of aptamer applications compared to the number of selections (Table 1). Here, some of the promising future applications are described (Figure 4). The challenges that must be addressed to make these potential applications a reality are summarized.

### 6.1. Diagnostics

The detection of DNA adducts poses a major analytical challenge due to their very low abundance in genomic samples. As a result, methods must be both very sensitive and highly specific [84]. DNA lesion investigations from the 1980s were typically accomplished using the ^32^P-postlabeling methodology. This method was capable of detecting lesions at frequencies as low as one lesion in 10^10^ nucleotides [85]. More recently, mass spectrometry (MS) coupled with liquid chromatography–electrospray ionization spectrometry (ESI-LC-MS) has been employed [86], and can currently be used to detect one lesion per 10^8^–10^9^ nucleotides. Regardless, these methods require significant quantities of purified DNA for quantifications. As a result, there have been several recent efforts toward specifically amplifying DNA adducts or mapping adducts within a genome. As an example, the Sturla group has developed a strategy to PCR-amplify DNA adducts of interest using mutated polymerases capable of specifically incorporating non-natural nucleosides [87]. As another example, the Burrows group has applied nanopore sequencing, and developed a biotin-labelling strategy to enrich and sequence oxidative damage [88,89]. These strategies are powerful, yet time-consuming and costly. Therefore, there is still an unmet need for simple, inexpensive methods that could be used as point-of-care diagnostic tools for decision-making about further DNA damage testing. Aptamers have been readily incorporated into nanoparticle- [90] and electrochemical-based [91] platforms to create point-of-care diagnostics. Therefore, the aptamers described in Table 1, could be easily used to rapidly screen or test for target DNA adducts.

### 6.2. Cellular Imaging

Immunofluorescent staining of DNA damage and the damage response has led to several important insights into DNA repair processes [92]. However, antibody staining must be performed after fixing of tissue or cell samples [93], and therefore cannot capture real-time or dynamic repair information. To image real-time processes inside cells, Jaffrey and co-workers developed “Spinach”, another breakthrough aptamer technology [94]. Spinach, and several newer variants, is an RNA aptamer capable of specifically binding to a small molecule dye. Only upon interaction with the RNA aptamer does the dye fluoresce, creating a “light-up aptamer”. These RNA aptamers have been encoded in the genomes of bacteria, yeast and mammalian cells to quantify, image and track specific RNA molecules in real-time [95,96]. More importantly, the Spinach aptamer can be directly coupled to a second aptamer, creating a system where fluorescence is observed only in the presence of the aptamer’s target [97]. As a result, metabolites have been imaged and quantified in live cells (see review [98]). Therefore, it is feasible that the same strategy could be applied to imaging and quantification of DNA adducts and repair proteins in live cells and whole animals.

### 6.3. Therapeutic Targets

With several candidates in the clinical pipeline, aptamers have gained therapeutic visibility [99]. Aptamers have been used to impair cancer development, inflammatory disease, viral infection and cardiovascular illness [99]. However, the most successful example continues to be Pegaptanib, a vascular endothelial growth factor antagonist aptamer which was approved in 2004 for treatment of age-related macular degeneration [100]. Recently, aptamer applications in therapeutics have focused on cancer drugs and targeted drug delivery [101]. The aptamers described in this review could be leveraged for these applications.

In particular, alkylating agents are commonly used in chemotherapy due to their ability to cause DNA damage-induced apoptosis [102]. However, the efficiency of chemotherapeutic agents is strongly reduced by DNA repair systems. Aptamers that bind to the active site of repair proteins or polymerases involved in repair could be used to specifically inhibit their activities in cells [54]. This is a particularly exciting application for aptamers, compared to antibodies, given that aptamers are sufficiently small to fit inside tight binding pockets [27]. As one example, the aptamer selected by the Krylov group, not only bound to the NER repair protein AlkB, but also was capable of efficiently inhibiting catalysis at nanomolar concentrations [31].

As another possible direction, aptamers could be used to target specific cells or “turn on” drug activity in the presence of altered repair processes. It is known that repair processes are altered in cancer cells and that DNA damage and defects in DNA repair can both cause cancer [103]. Certainly, aptamers that recognize cancer cell surface proteins may be useful in delivering DNA damaging chemotherapeutics or DNA repair inhibitors. Alternatively, typical mutated proteins arising from DNA damage (or lack of repair) may be useful for ensuring chemotherapeutics are successfully distributed and/or activated once inside their target cells (in this case, cells that indicate increased DNA damage or altered repair). For example, the aptamer that binds to the mutated p53 protein was only able to interact with the mutated protein in cancer cells in vitro as well as in tumor xenografts [62], and had no impact on the wild-type p53 protein. Therefore, it is feasible that aptamers that bind to mutated gene products could be loaded with drugs that target repair processes, providing a means to target cells that are either cancerous or that may lead to further damage and mutation.

### 6.4. Application Roadblocks

Despite the exciting potential, it is clear that there have been very limited demonstrations of using aptamers in DNA damage and repair applications. This lack of utility is likely due to several technical and conceptual challenges. For diagnostic applications, perhaps the biggest roadblock is that there is still much to learn concerning the quantitative importance of DNA damage. For example, the linear dose response for genotoxicant-induced gene mutations and chromosomal damage has been challenged [104]. Furthermore, there is a gap in our understanding of the link between sequence-specific DNA adducts and mutational patterns [56,105,106]. Until these questions are addressed, it is not clear if there is a need for rapid point-of-care diagnostics or testing DNA adducts as a biomarkers in disease.

In contrast, cellular imaging with aptamers is a much-needed application, particularly in the field of DNA damage and repair. However, this application has only been very recently demonstrated. It is likely “only a matter of time” before in vivo or cellular imaging of DNA damage and repair processes with aptamers are demonstrated. One challenge is the internalization of the cellular imaging probes. However, researchers have developed many clever transport and delivery strategies to overcome this hurdle (see review [98]), and thus it is no longer a critical issue. However, a bottleneck that has not been tested with this set of aptamers is the in vivo activity. Unfortunately, many of the available aptamers were selected in conditions that are not physiologically relevant. In particular, many aptamers were selected using high concentrations of magnesium. This can be a limitation when genetically-encoding or delivering aptamers into the cellular environment, where free magnesium is much less abundant [107]. Therefore, it is possible that additional aptamer selections, under physiologically relevant conditions, may be needed to enable the successful use of aptamers in cellular imaging of DNA damage and repair.

The majority of the aptamers selected for DNA damage and repair proteins used proof-of-concept targets, including prokaryotic versions of repair proteins. These aptamers would not be useful for therapeutic applications (as they likely do not bind to the human orthologues), but demonstrate the potential for future aptamer selections targeting human repair proteins. In general, the main challenge in applying aptamers that recognize DNA damage and repair targets to therapeutic applications are the same obstacles for all aptamer therapeutic targets. These challenges include efficient internalization of the aptamers into the cell (and nucleus), off-target binding, half-life and stability in vivo and renal clearance. These difficulties, in combination with a general reluctance to divert from conventional antibody-based approaches, account for the long delay in the clinical translation and distribution of therapeutic aptamers [99]. As the number of aptamer modifications increases, these challenges will no longer hinder the therapeutic use of aptamers in general. Subsequently, it is expected that the therapeutic applications for aptamers targeting DNA damage and repair processes will also improve.

## 7. Conclusions

There are few examples of aptamers that bind to DNA adducts or modified nucleic acids, damage repair proteins and mutated gene products involved in carcinogenesis. However, there are many targets for which future aptamer selection would be beneficial. For example, there are no aptamers that bind to damage resulting from deamination or crosslinking. Furthermore, aptamers could be selected for a long list of repair proteins, including those involved in the NER and HR pathways. Despite the potential, direct evidence of the utility of aptamers in the field of DNA damage and repair is currently limited. Therefore, demonstration of these exciting potential applications should be performed. It is expected that several ongoing advances in the field of aptamers, particularly in the context of targeted therapeutics, will help address current challenges limiting the applications of aptamers in DNA damage and repair. Ultimately, this may motivate the researchers to undertake additional selections to increase the list of aptamers that bind to DNA adducts and repair proteins.

## Figures and Tables

**Table 1:** Aptamers for DNA damage and repair targets.

Target Class	Target	Nucleic Acid	*K*_d_^1^	Reference
DNA Adducts	8-oxodG	RNA	270 nM	[16]
	8-oxodG	DNA	100 nM	[17]
	8-oxoG	DNA ^2^	5.5 µM	[19]
	8-oxoG	DNA	3 nM	[21]
	8-oxodG	DNA	25 µM	[21]
	(−),-(*R*)-dSp	DNA	28 nM	[21]
	(+),-(*S*)-dS	DNA	76 nM	[21]
	(−),-(*R*)-Sp	DNA	12 nM	[21]
	m^7^-GTP	RNA	500 nM	[22]
	benzylguanine	RNA	200 nM	[23]
Strand Breaks	homopurine/pyrimidine duplex	RNA	1 µM	[24]
	20 bp duplex	DNA ^3^	43.9 nM	[25]
	3′LTR	RNA	300 nM	[26]
	Ku protein	RNA	2 nM	[27]
Repair Proteins	Fpg (DNA glycosylase)	RNA	2.5 nM	[28]
	Polβ/polκ	RNA	290 nM	[29]
	MutS	DNA	3.6 nM	[30]
	AlkB	DNA	20 nM	[31]
	AlkB homologue 2	DNA	85 nM	[32]
Mutated Gene	KRAS^V12^	RNA	4.04 nM	[33]

^1^ Only aptamers with *K*_d_ values are reported; for each, the best *K*_d_ is included. ^2^ With a β-alanine side chain. ^3^ Presence of benzoindoloquinolin required.

## Abbreviations

ABH2	AlkB homologue 2
AGT	*O*^6^-alkylguanine DNA alkyltransferase
BER	Base excision repair
BIQ	Benzoindoloquinoline
bp	Base pair
DSB	Double-strand break
dsDNA	Double-strand DNA
EMSA	Electrophoretic mobility shift assay
Fpg	Formamidopyrimidine DNA glycosylase
GA	Glycidamide
HR	Homologous recombination
*K*_d_	Dissociation constant
LTR	Long terminal repeat
m^7^-GTP	7-methylguanosine 5′-triphosphate
NECEEM	Non-equilibrium capillary electrophoresis of equilibrium mixtures
NER	Nucleotide excision repair
NHEJ	Non non-homologous end joining
N7-meG	*N*^7^-methylguanine
8-oxoG	8-oxoguanine
PNA	Peptide nucleic acid
SELEX	Systematic evolution of ligands by EXPonential enrichment
Sp	Spiroiminodihydantoin
